# Impact of COVID-19 on the essential healthcare services at primary healthcare level: Serine Sahakyan

**DOI:** 10.1093/eurpub/ckac131.064

**Published:** 2022-10-25

**Authors:** V Hayrumyan, A Abrahamyan, A Harutyunyan, S Sahakyan

**Affiliations:** Turpanjian College of Health Sciences, American University of Armenia, Yerevan, Armenia

## Abstract

**Background:**

The COVID-19 pandemic triggered numerous challenges for the healthcare systems worldwide, particularly affecting the continuity of essential health services in low- and middle-income countries. We explored the effects of the COVID-19 pandemic on the utilization and delivery of essential health services in Armenia.

**Methods:**

We applied a conventional qualitative study design using semi-structured in-depth interviews (n = 17) in public and private primary healthcare (PHC) facilities in Armenia (2021). Participants included physicians providing primary health services (e.g. endocrinologists, gynecologists/obstetricians, and pediatricians), regular PHC facility patients (e.g. adults with chronic diseases, parents of children), and policymakers. Iterative thematic analysis was done based on inductively emerged 3 main themes: patient-provider communications, maternal and child health services, and management of chronic diseases.

**Results:**

Overall, visits to the PHC facilities were decreased due to fear to contract COVID-19 coupled with lack of information, misinformation and panic. There was a lack of digital platforms for ensuring continuous patient-provider communication and phone calls were the main way of communication. PHC providers intentionally limited the number of maternal and child visits to only essential antenatal visits, newborn screenings and routine child immunizations. Still, the latter has suffered resulting in delayed and decreased vaccinations. The pandemic remarkably decreased the number of follow-up visits and monitoring of patients with chronic conditions resulting in more critical and severe conditions.

**Conclusions:**

The COVID-19 pandemic affected the provision and utilization of essential health services at PHC facilities by changing people’s health-seeking behavior. Unified national-level guidance for PHC facilities is needed to direct the provision of essential services, effective health communication and usage of digital platforms.

**Key messages:**

• Though provider encounters should be limited during outbreaks, continuous provision of essential services is critical in the prevention of morbidity, complications and worsened disease severity.

• Efforts are needed to develop effective health and risk communication strategies and enhance appropriate usage of digital platforms to promote adequate health-seeking behavior among the public.

